# Raloxifene Improves Cognition in Schizophrenia: Spurious Result or Valid Effect?

**DOI:** 10.3389/fpsyt.2017.00202

**Published:** 2017-10-12

**Authors:** Thomas W. Weickert, Cynthia Shannon Weickert

**Affiliations:** ^1^School of Psychiatry, University of New South Wales, Sydney, NSW, Australia; ^2^Neuroscience Research Australia, Sydney, NSW, Australia

**Keywords:** schizophrenia, cognition, raloxifene, selective estrogen receptor modulator, symptom severity

Schizophrenia is a heterogeneous disorder characterized by psychotic symptoms and disabling cognitive deficits. The cognitive deficits may appear early in the illness prior to psychosis and are related to functional disability. Currently, there are no approved treatments to restore cognitive abilities in schizophrenia. Thus, there is an urgent unmet need: to reverse cognitive impairments in people with schizophrenia which may also ameliorate social and vocational difficulties.

Despite many failed clinical trials of cognitive treatments in schizophrenia, there are some potential candidates. Four clinical trials, including one by our own group, have reported the effects of the selective estrogen receptor modulator (SERM) raloxifene as an adjunctive treatment for symptom severity reduction and cognitive restoration in schizophrenia (1–4). Two (1, 2) out of these four studies have shown cognitive benefits of adjunctive raloxifene treatment. In one study, oral administration of 60 mg daily raloxifene treatment improved memory and executive function in postmenopausal women with schizophrenia (1) and in our study, oral administration of 120 mg daily raloxifene improved attention and verbal memory in men and women with schizophrenia (2). In a series of three additional reports based on patients who were part of our clinical trial showing a benefit of 120 mg daily raloxifene on verbal memory and attention in schizophrenia (2), we found that groups of 20–30 people with schizophrenia showed increased brain activity (cortex and hippocampus) during raloxifene treatment while performing different cognitive tasks (probabilistic association learning, emotional face recognition, and emotional word inhibition) (5–7). However, two studies did not demonstrate cognitive benefits of orally administered, 120 mg daily raloxifene treatment in postmenopausal women with schizophrenia (3, 4) casting some questions on the generalizability of the beneficial effects. Here, we critically assess some potential factors for discrepant results among these trials in relation to the effects of raloxifene administration on cognition in schizophrenia.

As to why these study results may differ, Kulkarni and colleagues (3) suggest that “mixed sex analyses may not demonstrate the actual potential of hormone treatments such as raloxifene.” This interpretation is unclear given that in our work (2) a mixed sex analysis revealed that oral raloxifene administration at 120 mg daily improved verbal memory and attention in both men and women with schizophrenia. Given that a raloxifene effect was demonstrated in a mixed sex sample, we suggest several alternative considerations as potential key differences among these raloxifene studies which may account for the discrepant findings.

Comparison of the raloxifene trials reveals several design differences. While the cognitive tests used in Weickert et al. (2) and Kulkarni et al. (3) were not identical, the same cognitive domains (e.g., immediate and delayed verbal memory) using very similar assays (i.e., story/paragraph recall also known as logical memory) were assessed in both trials yielding different results: raloxifene benefits on verbal memory and attention in Weickert et al. (2) and a failure to find raloxifene benefits in Kulkarni et al. (3). Although the Brief Assessment of Cognition in Schizophrenia battery (8) used in Weiser et al. (4) included list learning as a measure of verbal memory (which may be more dependent on working memory) and symbol coding as a measure of attention, which were different tests than those used in Weickert et al. (2) and Kulkarni et al. (3), these tests are generally thought to assess the same cognitive domains measured in the other raloxifene trials. Thus, in one comparison (2), versus (4), the tests were somewhat different while in the other comparison (2), versus (3), the tests were more similar, yet both comparisons of the tests used in these trials assessed similar cognitive domains, but yielded different effects of raloxifene on cognition in schizophrenia. Given the similarity between the tests used in Ref. (2) versus (3), it seems unlikely that test differences would account for different outcomes among the studies.

While other studies used a parallel group design (1, 3, 4), we used a cross-over design (2) [for further discussion on the advantages and disadvantages of between-subjects/parallel groups versus within-subjects/cross-over designs see Ref. (9)]; however, we also analyzed our data at 6 weeks of treatment (before the cross-over) using a parallel group design analysis. Our study (2) recruited both sexes, whereas other null effect trials (3, 4) only assessed postmenopausal females. In the event that younger men with schizophrenia respond more robustly than older women with schizophrenia to the cognitive-enhancing effects of raloxifene, a male only or a mixed sex analysis (rather than a female only analysis) could be advantageous toward finding an effect. Another factor pertains to the number of participants evaluated. In our parallel group analyses, we had a relatively larger sample (raloxifene *n* = 40, placebo *n* = 39) (2) than Kulkarni and colleagues (raloxifene *n* = 26, placebo *n* = 30) (3), but a smaller sample than Weiser and colleagues (raloxifene *n* = 92, placebo *n* = 85) (4). The Kulkarni and colleagues sample size (3) may have been insufficient to detect significant raloxifene effects on cognition; although Huerta-Ramos and colleagues (1) found raloxifene-related cognitive benefits in a small sample of postmenopausal women with schizophrenia (raloxifene *n* = 16, placebo *n* = 17), while Weiser et al. (4) did not obtain a raloxifene benefit in a substantially larger sample of postmenopausal women with schizophrenia. Thus, overall these findings suggest that analysis and sample size factors may not account for differences in trial outcomes.

Other important considerations are related to dose (60 mg/day versus 120 mg/day) and duration of treatment. Other trials showing no raloxifene effects on cognition administered 120 mg/day of raloxifene for longer durations (12 or 16 weeks) (3, 4) than our trial in which raloxifene was administered at 120 mg/day for 6 weeks (2). In contrast to other longer duration treatment trials showing no cognitive benefit following 12 or 16 weeks of raloxifene treatment (3, 4), Huerta-Ramos and colleagues reported cognitive benefits of 60 mg daily raloxifene after 12 weeks of treatment (1). A longer treatment duration may be more likely to reveal beneficial effects; however, our trial showed sustained cognitive benefits with a shorter treatment duration (2), suggesting that treatment duration differences alone are not likely to explain the differential raloxifene effects on cognition. However, a combination of treatment duration and dosage may explain the differences among trials, with a lower dosage over a longer duration or higher dosage over a shorter duration yielding beneficial cognitive effects of raloxifene.

Another important factor that could potentially explain differential cognitive effects of raloxifene pertains to the age of the participants. The mean patient age of 53 years (3) and 56 years (4) was substantially older than the mean age of 35 years old in our trial (2). Raloxifene administration in a younger sample may elicit a greater raloxifene cognitive response (2); although the Huerta-Ramos and colleagues trial did demonstrate cognitive improvement with raloxifene in older women with schizophrenia (1). Thus, similarities of participant age between the null effect trials (3, 4) and the Huerta-Ramos et al. (1) trial suggests that participant age may not contribute to the differential effects demonstrated among the studies.

Perhaps the most critical factor pertains to symptom severity scores at time of recruitment into each trial. Studies targeting symptom severity reduction usually recruit severely symptomatic patients (10). Trials that failed to show cognitive benefits of raloxifene had mean PANSS positive, negative, general and total symptom severity scores of: 18, 20, 40, and 78 (3), and 24, 27, 52, 103 (4), respectively, which were clearly higher than trials showing raloxifene cognitive benefits in which participants had substantially lower mean PANSS scores (15, 14, 31, 60) (2) and (10, 22, 30, 62) (1). Thus, it is the baseline symptom severity scores on which the trials that failed to show cognitive benefits of raloxifene (3, 4) and other raloxifene trials showing cognitive benefits (1, 2) specifically diverge, and it is the baseline symptom severity which we propose would be the key factor influencing the differential treatment effects among the trials. High symptom severity ratings would be expected to favor finding raloxifene effects on symptom severity reduction over cognitive benefits (10). In studies designed to test drugs for benefits on cognition, symptom severity (especially positive and general symptom severity) should not be acute and severe, but instead, symptom severity should optimally be constrained to a reduced and relatively stable level. It is important to note that even when the symptom severity is reduced, patients still display substantial and enduring cognitive deficits (2). However, minimizing symptom severity during the sample selection/recruitment process would impair the ability to detect symptom severity reduction with raloxifene (or other) treatments (10). In support of this key design difference, we found that raloxifene improves cognition but did not significantly influence symptom severity (2). Thus, these clinical trials of raloxifene in people with schizophrenia (1–4) generally found effects on symptoms and cognition in schizophrenia as would be expected [although one trial (4) failed to find significant symptom severity reduction], given the very important clinical differences (especially symptom severity at intake) that exist as outlined above. Therefore, these trials (1–4) may all be correct and both the reduction in symptoms and cognitive improvement could both be accurate and reproducible if similar methods and designs are used to replicate these trials. However, as noted, the evidence for raloxifene cognitive benefits in schizophrenia to date is somewhat mixed and not conclusive. Thus, further studies in larger samples, using baseline symptom severity to target appropriate (symptom or cognitive) outcome measures, would be warranted to replicate existing reports and identification of responder characteristics may be useful to address heterogenous treatment response.

Given careful screening/monitoring to prevent adverse events, raloxifene may be useful for either cognitive restoration or symptom severity reduction in schizophrenia, or possibly even both. In writing this critique, we hope to generate further hypotheses (e.g., regarding dosage, treatment duration, and baseline symptom severity levels) that can be tested to better determine the effects of raloxifene treatment on cognition in schizophrenia. Further research on estrogen and estrogen-based treatments (such as SERMs) may reveal other related compounds with the dual benefits for cognition and symptoms in schizophrenia. Given that the cognitive deficits of schizophrenia are related to functional disability and have been generally unresponsive to various treatments, a treatment such as the SERM raloxifene showing medium to large effect sizes of 0.64 (2) that can normalize performance and brain activity (5, 7) provides encouraging support for further studies of raloxifene to restore cognitive abilities in people with schizophrenia.

## Author Contributions

Both authors contributed to the conceptualization of the work and contributed to the writing and/or editing of the manuscript.

## Conflict of Interest Statement

The authors declare that the research was conducted in the absence of any commercial or financial relationships that could be construed as a potential conflict of interest.

## References

[1] Huerta-Ramos E Iniesta R Ochoa S Cobo J Miquel E Roca M Effects of raloxifene on cognition in postmenopausal women with schizophrenia: a double-blind, randomized, placebo-controlled trial. Eur Neuropsychopharmacol (2014) 24:223–31. 10.1016/j.euroneuro.2013.11.012 24342775

[2] Weickert TW Weinberg D Lenroot R Catts SV Wells R Vercammen A Adjunctive raloxifene treatment improves attention and memory in men and women with schizophrenia. Mol Psychiatry (2015) 20:685–94. 10.1038/mp.2015.11 25980345 PMC4444978

[3] Kulkarni J Gavrilidis E Gwini SM Worsley R Grigg J Warren A Effect of adjunctive raloxifene therapy on severity of refractory schizophrenia in women: a randomized clinical trial. JAMA Psychiatry (2016) 73:947–54. 10.1001/jamapsychiatry.2016.1383 27438995

[4] Weiser M Levi L Burshtein S Hagin M Matei VP Podea D Raloxifene plus antipsychotics versus placebo plus antipsychotics in severely ill decompensated postmenopausal women with schizophrenia or schizoaffective disorder: a randomized control trial. J Clin Psychiatry (2017) 78:e758–65. 10.4088/JCP.15m10498 28541645

[5] Kindler J Weickert CS Skilleter AJ Catts SV Lenroot R Weickert TW Selective estrogen receptor modulation increases hippocampal activity during probabilistic association learning in schizophrenia. Neuropsychopharmacology (2015) 40:2388–97. 10.1038/npp.2015.88 25829142 PMC4538353

[6] Ji E Weickert CS Lenroot R Kindler J Skilleter AJ Vercammen A Adjunctive selective estrogen receptor modulator increases neural activity in the hippocampus and inferior frontal gyrus during emotional face recognition in schizophrenia. Transl Psychiatry (2016) 6:e795. 10.1038/tp.2016.59 27138794 PMC5070055

[7] Kindler J Weickert CS Schofield PR Lenroot R Weickert TW. Raloxifene increases prefrontal activity during emotional inhibition in schizophrenia based on estrogen receptor genotype. Eur Neuropsychopharmacol (2016) 26:1930–40. 10.1016/j.euroneuro.2016.10.009 27842943

[8] Keefe RSE Goldberg TE Harvey PD Gold JM Poe MP Coughenour L The Brief Assessment of Cognition in Schizophrenia: reliability, sensitivity and comparison with a standard cognitive battery. Schizophr Res (2004) 68:283–97. 10.1016/j.schres.2003.09.011 15099610

[9] Martin DW Doing Psychology Experiments. 7th ed Belmont, CA: Thompson/Wadsworth (2008).

[10] Furukawa TA Levine SZ Tanaka S Goldberg Y Samara M Davis JM Initial severity of schizophrenia and efficacy of antipsychotics: participant-level meta-analysis of 6 placebo-controlled studies. JAMA Psychiatry (2015) 72:14–21. 10.1001/jamapsychiatry.2014.2127 25372935

